# Understanding the barriers and enablers for postgraduate medical trainees becoming simulation educators: a qualitative study

**DOI:** 10.1186/s12909-022-03995-3

**Published:** 2023-01-14

**Authors:** Albert Muhumuza, Josephine Nambi Najjuma, Heather MacIntosh, Nishan Sharma, Nalini Singhal, Gwendolyn L Hollaar, Ian Wishart, Francis Bajunirwe, Data Santorino

**Affiliations:** 1 Faculty of Medicine, Mbarara University of Science and Technology, Mbarara, Uganda; 2 Cumming School of Medicine, University of Calgary, Calgary, AB Canada

**Keywords:** Postgraduate, Simulation educator/facilitator, Enablers, Barriers, Uganda

## Abstract

**Introduction:**

There is increasing evidence that Simulation-based learning (SBL) is an effective teaching method for healthcare professionals. However, SBL requires a large number of faculty to facilitate small group sessions. Like many other African contexts, Mbarara University of Science and Technology (MUST) in Uganda has large numbers of medical students, but limited resources, including limited simulation trained teaching faculty. Postgraduate medical trainees (PGs) are often involved in clinical teaching of undergraduates. To establish sustainable SBL in undergraduate medical education (UME), the support of PGs is crucial, making it critical to understand the enablers and barriers of PGs to become simulation educators.

**Methods:**

We used purposive sampling and conducted in-depth interviews (IDIs) with the PGs, key informant interviews (KIIs) with university staff, and focus group discussions (FGDs) with the PGs in groups of 5–10 participants. Data collection tools were developed using the Consolidated framework for implementation research (CFIR) tool. Data were analyzed using the rigorous and accelerated data reduction (RADaR) technique.

**Results:**

We conducted seven IDIs, seven KIIs and four focus group discussions. The barriers identified included: competing time demands, negative attitude towards transferability of simulation learning, inadequacy of medical simulation equipment, and that medical simulation facilitation is not integrated in the PGs curriculum. The enablers included: perceived benefits of medical simulation to medical students plus PGs and in-practice health personnel, favorable departmental attitude, enthusiasm of PGs to be simulation educators, and improved awareness of the duties of a simulation educator. Participants recommended sensitization of key stakeholders to simulation, training and motivation of PG educators, and evaluation of the impact of a medical simulation program that involves PGs as educators.

**Conclusion:**

In the context of a low resource setting with large undergraduate classes and limited faculty members, SBL can assist in clinical skill acquisition. Training of PGs as simulation educators should address perceived barriers and integration of SBL into UME. Involvement of departmental leadership and obtaining their approval is critical in the involvement of PGs as simulation educators.

## Introduction

Medical simulation based learning(SBL) is an effective tool for learning but it requires adequate numbers of trained educators to conduct small group sessions. [1–4] In low resource settings like Uganda, medical schools are often understaffed and faculty trained as simulation facilitators are seldom adequate [5–11]. The postgraduate medical trainees (PGs) are often involved in Undergraduate Medical Education (UME) [12] and may also serve as simulation educators.

The PGs around the globe perform multiple roles during their training and these include being learner, researcher, clinical practitioner and as near-peer educators for their junior counterparts [13–15]. Near-peer teaching has been described as an education strategy where a trainee who is one or more years senior participates in teaching another trainee at a lower level of education. Near-peer teaching is perceived as beneficial to both the undergraduate medical students and their postgraduate colleagues [16]. For this to be efficient, some level of formal training in teaching skills and knowledge is required [17, 19]. While junior learners benefit from constructive feedback and safe learning environment, their senior counterparts get the preparation needed to become future medical educators [17, 20, 21]. Evidence indicates mutual benefits for the student tutor and the learner involved in simulation based near- peer teaching [22].

In Ugandan medical schools, PGs medical trainees consider their involvement in teaching undergraduate medical students as beneficial [16]. At Mbarara University of Science and Technology (MUST) medical postgraduates are already involved in teaching undergraduate medical students on hospital wards and tutorials. PGs spend 20% of their time on teaching activities and they provide 20–70% of clinical teaching for medical students [23]. Undergraduate students also acknowledge that postgraduate medical students contribute significantly to their learning [24–26]. When trained, postgraduates are able to provide similar quality of simulation based teaching as trained faculty members [27]. The benefits of near-peer teaching have not been sufficiently exploited in the arena of Simulation Based Learning (SBL) and yet there is evidence that this method of teaching contributes to acquisition of knowledge and teamwork skills in undergraduate medical and nursing students [28].

There is limited data on barriers and enablers to a medical school simulation program that involves PGs as educators in a low resource setting. There is a dearth in research providing evidence on the best practices for adopting postgraduate medical trainees as simulation educators for undergraduate medical students. Understanding the barriers and enablers will inform the design and implementation of simulation-based curricula as well as interventions for scale-up of SBL in Africa. This is anticipated to improve undergraduate students’ learning outcomes and may also address the concerns of human resources needed for the sustainability of SBL in the absence of external funding [29]. Having a pool of PGs simulation educators is envisioned to increase simulation utilization in medical training which could potentially positively impact patient safety and quality of care [30].

Therefore, the purpose of this study was to explore the barriers and enablers to engaging PGs as simulation educators for undergraduate medical students and identify key priority areas for consideration prior to implementation of the intervention.

## Methods

### Study design

We conducted an exploratory qualitative study between February and May 2021 at Mbarara University of Science and Technology (MUST) in Uganda.

### Setting

MUST is a public university is southwestern Uganda, located 240 km from Kampala, the capital. The faculty of Medicine at MUST offers undergraduate degree programs in medicine, nursing, pharmacy, medical laboratory science and physiotherapy; and master’s degrees in several clinical and basic science programs. The university has two clinical skills labs and one fully functional medical simulation center. The MUST simulation center was established in 2016. MUST and collaborators established the simulation center through the Simulation for Life (SIM for LIFE) Program. The MUST Simulation Center has two scenario execution labs, one debriefing room and office space. Simulations are run by a combination of faculty from the university and SIM for LIFE program. Medical Simulation has been integrated into the curriculum and timetabled for undergraduate Bachelor of Medicine and Bachelor of Surgery as well as Bachelor of Nursing programs.

### Study participants

The study participants included two categories. The first was PGs enrolled in any of the five major Masters of Medicine programs namely Pediatrics, Internal Medicine, Obstetrics and Gynecology, Surgery, and Emergency Medicine at the Faculty of Medicine- MUST. The second was MUST administration and managers. This category included Heads of Departments from four major disciplines where undergraduate medical students do clinical rotations (pediatrics, obstetrics and gynecology, surgery, and internal medicine). These are key stakeholders in the adoption of PGs as simulation facilitators for undergraduate medical students and are responsible for simulation time allocation. Some of them have already been exposed to simulation and as such would be able to share actual experiences on the anticipated barriers and enablers. The PG study participants were recruited through phone call to departmental postgraduate student leaders. These leaders then scheduled a meeting where the study was explained by the qualitative research specialist and consent was obtained from the willing participants. The university administrators and managers were approached individually and study procedures were explained and consent obtained when they accepted to participate.

### Sampling and data collection

We purposively selected postgraduate medical trainees from the five Masters of Medicine programs, as these are disciplines where undergraduate medical student rotations have the most contact hours to enable adequate interaction with the PGs. The PGs selection purposely included participants from all the years of study. We conducted in-depth interviews (IDIs) with the PGs. For administrators and managers, we purposively selected those influential in PG training and conducted key informant interviews (KIIs). Overall, the same tool was used across the three categories of respondents, but was modified to suit the respondent. For instance, postgraduate students had questions on cost of intervention removed as they would these would not apply to them.

A semi structured data collection tool was designed using the Consolidated Framework for Implementation Research (CFIR) tool. We adopted questions from the five domains of the CFIR. The domains are: Intervention characteristics, inner setting, outer setting, characteristics of individuals and process of implementation. [31] The designed questions were from the constructs that applied to the study. The CFIR is a well-known framework [32] and was chosen for its organizational perspective with respect to implementation compared to other frameworks which tend to focus on individual level changes. For all participants we explored motivation, acceptability, and feasibility to engage PGs as simulation facilitators for undergraduate medical students.

Data were collected from key informant interviews, in-depth interviews of senior PGs and focus group discussions amongst junior PGs. The senior PGs are those with some teaching experience and are usually in their second or third year of training. The junior PGs are those with no teaching experience are usually those in their first year of study. The senior PGs participated in in-depth interviews because they had been involved in teaching undergraduates using other methods like tutorials. We carried out focus group discussions (FGD) from the four major clinical departments to engage the junior PGs. The FGDs were used because they stimulate diverse opinions to generate a rich conversation. We used key informant interviews for university administration because this was a small group with considerable knowledge and experience with simulation and processes of adoption of teaching practices. We have de-identified the description of these participants for confidentiality. Data collection was carried out by a trained and experienced qualitative research assistant. The interviewers were not part of the investigators and were not known to the participants. All interviews were attended by a note taker who took field notes. We collected data until we reached the saturation point [33]. Both FGDs and interviews lasted between 30–60 min. All interviews were conducted in English and were audio-recorded using a digital recorder.

### Data analysis and coding

**Fig. 1:**
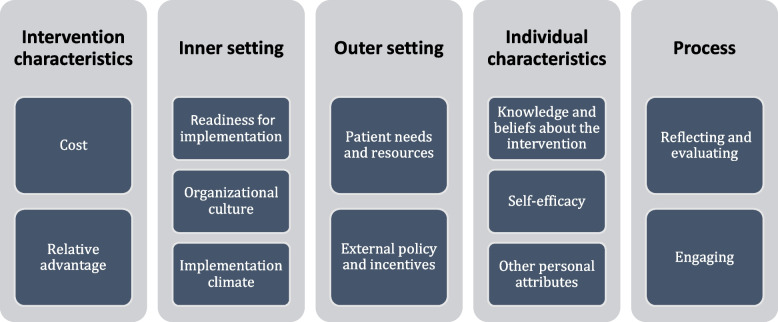
CFIR constructs for implementation of postgraduates as simulation facilitators

The audio files were transcribed verbatim and analyzed the data using thematic content analysis based on a priori themes generated from the research questions designed using the CFIR model and also considered emerging themes from the data (See Fig. 1 for CFIR generated themes). Analysis of the qualitative data started with the familiarization of the data through repeated readings of transcripts and reviewing of audio files. Codes were grouped into categories and condensed into broader themes. The first analysis was done by an independent qualitative research expert. AM and JNN double coded the transcripts and compared their results with that of the qualitative expert. Later, this team met with HM, NS, FB and SN to agree on the codes and resolve disagreements to generate final codes. RADaR (Rigorous and Accelerated Data Reduction) technique was used to analyze the qualitative data. RADaR is an individual or team based approach to code and analyze qualitative data where a data reduction process is undertaken to produce shorter and more concise tables [34]. We chose RADaR because it is recommended for small datasets.

### Ethical considerations

Research and ethical clearance to conduct the study was sought from the Uganda National Council of Science and Technology and Research (UNCST HS 2662) and the Research Ethics Committee of Mbarara University of Science and Technology (MUREC No 20/07–19). The study was conducted according to the international guidelines for ethical research as stipulated in the Helsinki Declaration. Administrative clearance to conduct the study was sought from the academic registrar of MUST. We obtained written informed consent from all the study participants. Privacy and confidentiality were maintained throughout the study. For in-depth interviews, key informant interviews, and focus group discussions, care was taken to ensure that all interviews are kept confidential. Each study participant was given a unique identification number written on the informed consent document. Only the participants’ identification number was included on the transcripts and audio files. All data was stored in password protected computer files and audio files were discarded after use.

## Results

### Demographics

We interviewed 7 university administrators and managers (5 males and 2 females) as key informants, 7 postgraduate trainees (6 males and 1 females) for in-depth interviews and 29 were involved in 4 FGDs. Three (3) postgraduate trainees were not able to join the focus group discussions due to scheduling challenges. (Table 1).

**Table 1:** Study participant characteristics

Data Collection Method	Study participants	Sample size
**Key-informant interviews**	Faculty administrators	7
**In-depth interviews**	Senior Postgraduate students	7
**Focus Group Discussion (FGD)**	PG students	29
	FGD 1	5
	Males	5
	Females	0
	FGD 2	6
	Males	2
	Females	4
	FGD 3	10
	Males	5
	Females	5
	FGD 4	8
	Males	7
	Females	1

### Interview and FGD results

#### Barriers to involvement of PGs as medical simulation educators

The barriers presented by the participants included: demand on time, negative attitudes towards transferability of simulation learning, inadequacy of simulation equipment and the lack of integration of PG facilitation of medical simulation into the curriculum. (See Table 2 for summary of results from qualitative interview data analysis).

**Table 2:** Understanding barriers and enablers of proposed intervention in line with the CFIR model

CFIR Domains Construct name			Thematic areas	Key points
**Intervention characteristics**	Relative advantage			Learners do not have enough access to patients on some scenarios during normal teaching sessions on wards. Simulation allows learners to bridge this skills gap
	Cost		Perceived barriers to implementation	There is concern whether the tools(manikins) for conducting simulation sessions are sufficient
**Outer setting**	Patient needs and resources		Understanding of medical simulation	Acknowledging simulation as one way to address patient safety
	External policy and incentives		Key priority areas	The need for engaging higher authorities such as the ministries to gain approval and improve acceptability
**Characteristics of individuals**	Knowledge and beliefs about the intervention		Perceived benefits of PGs as simulation educators	Enhances near peer teaching by providing; -Hands on skills -Confidence when managing a case scenario
			Attitudes towards simulation	May not give the actual picture as the real life scenario
			Key priority areas	Training simulation facilitators
**Inner setting**	Implementation climate	Compatibility	Anticipated barriers Enablers	Scheduling of simulation sessions versus other competing demands for both post graduates and undergraduate students Flexibility to change-some departments are keen to embrace innovations in medical education
		Tension for change	Perceived benefits of SBL	Current teaching limits practical opportunities for students thus the need for simulation
		Organizational incentives and rewards	Key priority areas	Certification of simulation facilitators
	Readiness for implementation	Leadership engagement	Key priority areas	The need for engaging heads of departments, faculty administrators and other university administration
		Available resources	Key priority areas	Ensure there are sufficient teaching and learning materials Space for conducting sessions Schedule simulation sessions into existing timetable
	Culture		Enablers	Departments are adaptable to change
**Process**	Engaging	Opinion leaders	Key priority areas	Bring the heads of department on board through sensitization
	Reflecting and evaluating		Key priority areas	Concern as to whether there will be an undergraduate medical student comparison group that did not receive the intervention

#### Competing time demands

Postgraduate medical trainees anticipated that the availability of time would be the biggest constraint to becoming simulation facilitators given their busy schedule and heavy workload. Their daily schedule is occupied with activities such as patient care in the hospital, lectures/tutorials and personal study. They emphasized that medical services in the hospital are generally overseen by the PGs due to the shortage of health workers. Therefore, participants thought involving PGs as medical simulation educators would be overwhelming and time consuming since the undergraduate students have to be divided into multiple groups.

*“Maybe the biggest weakness since we came here is time. We are students but at the same time we are medical practitioners. Our biggest challenge is that we are overwhelmed by clinic work. The hospital does not have medical officers, so it is our duty to oversee the medical services. So sometimes we may work the whole day or night. So, the biggest problem will be to find time to train these students” (Mixed FGD #3).*

*“Occasionally when we get here, we have to do some presentations and exams. So if I instead come to train students the following day and the day after, I would be distracted for a day. I am worried about how I will pass and you know that the pass mark is 60. It is painful to fail as an adult. It can be so embarrassing and inconveniencing” (IDI Male PG student #04).*

*“So me I think it’s going to be too much work for the postgraduates because they already have a lot. The best people would be if the numbers were very adequate, the lecturers of these students should do so.” (KII Admin 02).*

*“The way I am seeing it is that maybe lecturers have failed to give simulation and now plan B is to engage postgraduate students. But I see that as a wrong thing because postgraduates are here to learn and if they are to teach, it should be under the supervision of their lecturers…if you are taking post graduates, we should also be there” (Male Key informant #4).*

#### PG simulation education is not integrated in the curriculum

Some respondents viewed the engagement of PGs as simulation educators to be a research project. On the other hand, others expressed uncertainty as to whether medical simulation was officially taken up by MUST as a teaching method. Participants were concerned about whether simulation would fit into the existing curriculum due to time constraints and other competing demands. Consequently, this implied that some educators may not take it seriously as they may not consider it to be part of their routine activities. Experiences of how the intervention was already interfering with the curriculum were cited in these narratives;

*“It sometimes interferes with the normal curriculum or what they are supposed to do on a daily basis. For example, the other time, they asked me to send some postgraduates to become facilitators and they were supposed to spend full days. If I had to send first years yet they are doing basic sciences like anatomy and physiology, but spend full days at simulation, they may miss the important lectures for which they are going to be examined and if they fail, it is going to be bad in their side, they could even retake” (Male KII HOD #2).*

*“Now, this problem is, this program of medical simulation is very good but the problem is that I don’t know when it was being rolled out in this University” (Key informant #2)*

*“People need to know that simulation is part of the university program. I know that there are people that know that simulation is a project and therefore not part of the university program. So when you have people with such a mentality, they will not come think that there are specific people that are benefiting from the project” (Male Key Informant#5).*

#### Skepticism towards realism in medical simulation

Some participants exhibited skepticism towards transferability of simulation-based learning. They mentioned that simulation denies learners the opportunity to have the personal touch with the patient when in a simulation session. For some participants, there was an expression of doubt about whether some scenarios could be simulated because the scenario has to be realistic scenarios for learning to take place. In addition, being a novel intervention, some had doubt as to whether the intervention would work. One participants said,

*“If you are simulating to deliver a baby, there are a lot of things that can happen during the process which cannot be simulated (Mixed FGD #2)”.*

*“A dummy is far much different from a human being. I might be touching a dummy, but when I touch the skin of a human being, it is different …that means that it would not feel exactly like I am treating a human being (Mixed FGD #2).”*

#### Inadequacy of medical simulation equipment

Some participants were concerned about the adequacy of the training equipment including manikins in comparison with the number of learners to be taught.

*“You find that they want you to conduct a session but you do not have the exact numbers that you are supposed to use or they are not there at all. In my experience, I have seen one where we have one dummy and it was not enough for all of us” (Mixed FGD, #2).*

#### Enablers to PG involvement as simulation educators

Participants identified enablers to PG involvement as medical simulation educators for the UME to included; perceived benefits of medical simulation to students and in-practice personnel, favorable departmental attitude, enthusiasm to participate in simulation and the awareness of the educator role.

#### Perceived benefits of medical simulation

Respondents mentioned benefits of simulation-based learning to undergraduate and postgraduate medical students as well as in-practice medical personnel. For undergraduate students, medical simulation allows mastery of clinical skills as well as building knowledge, confidence and teamwork skills which could help students smoothly transition from theory to hands-on practice. Participants noted that with increasing number of medical students and increase in student-patient ratios, students have a reduced opportunity to work with real patients. Furthermore, trainers on wards provide limited practice opportunities because they undermine learner’s ability to handle some clinical scenarios. As a result, some students were likely not to have the opportunity for hands-on skills in managing certain medical cases, instead relying on theoretical knowledge acquired in lectures for the development of skills.

*“To me, I believe learning is by repetition. The more you do something, the more you understand it”. (FGD PG student # 4).*

*“Actually it’s really beneficial, it may not be exactly what happens in reality but at least it gives me a sense of direction of what to do when you get into the real world. Instead of just reading these things and looking at pictures, at least you learn, it is like mostly hands on” (IDI PG student # 1).*

*“It's (simulation) is a good learning tool. It is different from the conventional teaching methods that we know. I think, it will change the medical school a lot. You know there are medical students who go through and they have never even touched a patient” (IDI Female Postgraduate student #1).*

*“Well undergraduates are exposed to patients but how much they can do is a bit limited. For example, if I have a baby that is asphyxiated, it is very unlikely that I will trust the undergraduate to handle the situation. I can only allow them to observe. However, with simulation, they can have the hands on experience thus building their confidence especially when demonstrating their skills to more senior people” (Mixed FGD #2).*

*“however, with the simulation, you can organize them in small groups and you’re sure that every student has been able to have hands on, on that specific thing that you are going to teach which will not happen on the patient.” (Mixed FGD #1).*

*“Yeah, the advantage will be, you learn how to work as a team because you won’t always be working alone, and team work is the best way” (IDI Female PG student #3).*

Participants noted that while facilitating medical simulation sessions, PGs are able to efficiently teach undergraduates in small groups and in turn master their clinical skills too.

*It is a good idea, you know, when you teach, you also learn on the process, it helps you to improve yourself. (Mixed FGD #1)”*

*“Actually I believe that it is very advantageous to not only the undergraduates but also the post-graduates themselves. In my short experience, I have learnt that the more I teach others, the more I retain the same information and you know that majority of the contact time of undergraduate students is with residents” (IDI Male PG student #05).*

*“To me its awesome, I love simulation, I’ve been a beneficiary before and I think it’s a good thing, given our circumstances that our wards are crowded and undergraduates are very many, I think it would be an alternative to our clinical teaching generally.” (Mixed FGD #4).*

*“Given the terms for these medical students on ward, not every one of them has the opportunity to manage a case under your supervision because of the student numbers. However, with the simulation, you can organize them in small groups and you are sure that every student is able to have hands on with a given scenario (Mixed FGD #2).”*

Participants also mentioned that medical simulation was important in standardization of practice and continuous medical education for both nurses and doctors in practice.

*“I think for me simulation is a big thing and its where we are going because if we are going to develop then we are going to take it on because I’ve been reading those key areas and simulations have actually helped people, it is even good for doctors who have finished medical education, they go back to remind themselves of these specific things. Me I really love what simulations are really like.” (Mixed FGD #4).*

*“Simulation is good because you work stepwise. Use this one, if it fails, use this one. So it sharpens your knowledge before you face a real life scenario (IDI Male Postgraduate student #3).*

#### Favorable departmental attitude

Participants expressed a favorable departmental attitude pointing to the fact that PGs being simulation educators would not interfere with their educational schedules. It would instead enhance already existing teaching mechanisms in the different departments and would be a mere extrapolation of the teaching role postgraduates are already handling at the department level but would need some internal arrangements. Other participants acknowledged that their departments are always adaptable to change and therefore did not anticipate any challenges with accepting PGs as simulation facilitators.

*“Now that they are already teaching, it’s not anything wrong. Am very comfortable with that, after all we’ve been assigning them tutorials and other ward teachings so it’s not an extra burden to say, but rather extrapolating the scope what they have been covering so I have no objection…” (KII HOD #01).*

*“It’s to a good extent, people are so flexible especially when there are innovations or ideas that will make change, or in patient care, they are embraced…but it obviously depends on what idea it is, what are the intended goals to know how to address them” (KII HOD #3).*

*“Things are always advancing. From my experience in the pediatrics department, they always want what works best for the child…we always go an extra mile that other departments may not go (IDI Female PG student #1).*

#### Enthusiasm to participate in simulation

Post graduate medical trainees were enthusiastic to become simulation facilitators given that they were already engaged in teaching undergraduate medical students using other methods. Both PGs and university managers acknowledged that PGs spend significant time with undergraduate learners. This presents an opportunity for near-peer teaching on ward or even in the simulation center. They noted that one of the expectations of PG students is to teach and part of their assessment is how they teach. Furthermore, PGs who had preexisting passion for teaching saw it as an opportunity to learn a new skill.

*“Bottom line, in every department, it is the post graduates who spend the most time with the undergraduates, they are the ones who teach them…I think that they will take this (simulation facilitation) (Male Key informant #1).”*

*“I think us being Simulation Facilitators for undergraduates would be a good idea. I believe, we are the people who are with them all the time on ward. And we understand every other person’s weakness, actually.” (IDI PG student #2).*

*“Now that they are already teaching, I am very comfortable with that. After all, we’ve been assigning them tutorials and other ward teachings, so it’s not an extra burden but rather extrapolating the scope of what they have been covering” (Male Key informant #2).*

*“If I am to look at it from my perspective, whenever the seniors are coming to do simulation with the undergraduates, they are always accompanied by the postgraduates on a daily basis. There has not been a day when I came here to conduct simulation without undergraduate students. This implies to me that the PGs are willing to take this up” (Male PG student #2).*

*“I told you, I’ve never seen what is in simulation, meaning that I will acquire a skill and that is a personal benefit” (Mixed FGD#3).*

#### Awareness of the duties of a simulation educator

Participants identified the simulation educator as an individual who has sufficient knowledge to guide learners through a scenario while identifying mistakes made by the learners and thereafter corrects the mistakes or confirms what they have done right. The educator can also demonstrate to learners how that scenario should be approached. Participants mentioned other duties and these included: 1. Ensuring that the simulation process had been done from the start to the end and a report of the simulation facilitation exercise recorded 2. Securing a venue for conducting a simulation session and making sure that all the necessary equipment was available before the session starts, 3. Giving students the opportunity to perform the procedure themselves, assessing the students and giving accountability and 4. Developing scenarios that are common in the specific departments so that students learn how to manage these cases. Additionally, they mentioned that the facilitator should be amiable given that they are mostly dealing with a younger audience. Some respondents noted that an unfriendly facilitator may impede learning during the simulation session. Participants mentioned that:

*“I think it is very important for the facilitator to secure a venue where the simulation is going to take place and then also making sure there are necessary equipment, for this process to go on, before we even go into the real thing.” (Mixed FGD-SIM PG #).*

*“Well that would be to teach but they can also design the scenarios based on what is common and that could be good or could vary from one person to another. That may not be the same as that of obstetrics or internal medicine so they can come up with scenarios for which they want to teach” (KII-Admin 01).*

*“Some facilitators are very rough such that when you ask them a question, they will be like “eh, you don’t know that?” (Mixed FGD #1)*

Participants also identified key priority areas that need to be considered before PG involvement in simulation based undergraduate medical education. These included sensitization and engagement of departments in simulation activities, training and motivation of PG facilitators and evaluation of the impact of the PG facilitated simulation learning. These are further explained below.

#### Departmental sensitization and engagement in simulation activities

Participants mentioned that there was need to engage the department heads or seniors. They emphasized a need for proper communication to the department about the benefits of simulation, plans to engage the PG facilitators and their proposed roles. They mentioned this would inform department scheduling processes to help to address the issue of time constraint and avoid clashing with other programs. Respondents also mentioned that senior department members could help support PGs since they have more experience in supervising the simulation sessions and should therefore be involved in the initial training of PGs as simulation facilitators.

*“…they will not accept because the best is that simulation center is to come to the department and enlighten, it’s to roll out PG simulation facilitation program and tell people what simulation is all about, and the procedures and then it will be easy for the head of department to say; “I am nominating two people to go” because some people don’t know whether simulation center is in the department” (Key informant HOD#02).*

Some suggested that it should be tailored to departmental schedules individually because they are usually different.

*“I believe, that this program should be tailored to the individual department, like a universal approach because it surely wouldn’t work. Because even as residents from different departments, we have different work schedules” (IDI PG student #1).*

#### Training and motivation of PG simulation educators

Participants acknowledged the need for simulation facilitators/educators to undergo foundational training so that they can gain full knowledge of the implementation process of the intervention, gain uniform facilitation skills and overall knowledge of medical simulation. However, they differed in the mode of training that would be most convenient. Some opted for a partly online course to address time constraints while others thought a purely in-person physical training would be most efficient. Participants suggested that the PGs should also accrue some benefit from being simulation facilitators. Such benefits included certification that is accredited or recognized while others expected remuneration as an incentive to conduct the simulation sessions.

*“*Otherwise, putting us into the program as simulation facilitators is a good idea for undergraduate students to learn as long as we are well trained to be good facilitators” (Male PG student #2).

*“So you come here, not getting marks, not getting any reward, it will not arouse any interest in view of the long term” (IDI Male PG student #07).*

#### Evaluation of the impact of PG facilitated simulation learning

Respondents proposed a comparison of undergraduate medical students who had been exposed to simulation by PGs and those who had not. They thought that would help to understand whether a program that involves PGs as simulation educators is impactful or not. Participants also proposed the use of medical simulation in clinical assessment for undergraduate medical students to detect changes in student performance and to encourage adherence to the program.

*“As undergraduates, we did simulation but did not take it seriously, students would only play with the dummies and end there. So I think there is need to make simulation examinable so that learners can take it serious (IDI Male PG student #02)”*

## Discussion

This paper explores the barriers and enablers to adopting postgraduate medical students as simulation facilitators as well as key areas for considerations when implementing a ‘postgraduate as teacher’ simulation program in a low resource setting. Our study identified significant barriers such as time constraints, perception that medical simulation was research and skepticism about transferability of skills to actual patients since the demonstration of SBL is done using manikins.

### Barriers

The PGs mentioned demand on time as a major constraint to participation as simulation educators. As anticipated, they spend significant amount of their time dedicated to patient care, teaching undergraduate tutorials, and personal reading/lectures according to the curriculum. Some university administrators and Heads of Departments (HoDs) were concerned that adding the role of simulation educators to PG medical students would be overwhelming given the constrained PG schedule. However, other HoDs saw this as merely an extension of the already existing PG teaching role in UME. Lack of time and adequate ward cover has been highlighted in prior studies as barriers to involvement of medical PGs as near-peer teachers for junior colleagues [35–37]. PG participation as simulation facilitators from a previous study was enabled by the scale up of a voluntary program into a mandatory longitudinal simulation program which in turn facilitated sustainability [38]. Miloslavsky et al. embedded a medical simulation-based Resident-as-Teacher (RaT) program in an already existing simulation curriculum for medical interns. This integration was well received by both the interns and PGs [23]. This observation strengthens the need to consider effective timetabling as an enabler to creating dedicated time for PG simulation involvement. Blending medical simulation facilitator roles into the expectations of a PG could enable their involvement as demonstrated in other methods of teaching [39].

Medical simulation was viewed as a research project rather than a university program. This presents a barrier to engaging medical PGs as simulation facilitators for undergraduate students if viewed as a project with a life span. Research programs are largely perceived as separate or external entities and if simulation is placed in this bracket, it may discourage participation and affect integration into institutional activities [40]. The term ‘research project’ communicates transient engagement which may not be considered part of routine activities in an institution [40]. The involvement of stakeholders such as decision makers and researchers in the design and conduct of interventions has been emphasized as important in improving perceptions [41, 42]. Engaging departmental stakeholders in planning and execution of medical simulation program activities can achieve interdisciplinary involvement and ownership.

Some participants were skeptical towards the transferability of SBL because they felt manikins did not reflect reality. These perceptions have been described among traditionalists who place high value to face to face learning [43, 44]. For successful adoption of SBL involving PGs as facilitators, it is important that this intervention fits the values, and beliefs of key stakeholders [41]. Changing perceptions and beliefs is a gradual process [45]. The champions of medical simulation need to assist the traditionalists to see benefit of medical simulation in enhancing skills and knowledge acquisition as well as critical thinking [43]. Educating faculty and stakeholders on the best practices of medical simulation facilitation has been shown to prove to skeptics the productiveness of this teaching method [46]. This in turn achieves buy-in and results in creation of more learning experiences for the students [46].

### Enablers

The perception of SBL as beneficial coupled with favorable departmental attitude towards medical simulation are significant driving factors. Participants mentioned benefits to both undergraduate and postgraduate medical trainees as well as in-service health workers. For the undergraduate medical students, participants highlighted: 1. allowing repetitive practice without life at stake, 2. creating a smooth transition from theory to practice and 3. creating opportunity for small group supervised hands-on practice which is becoming increasingly rare as medical student numbers grow. Other values like building confidence and teamwork skills have been mentioned in previous studies [28, 47, 48]. The heads of department and PGs anticipated that there would be benefits to the PGs too. These benefits included improving teaching skills and retention of knowledge and clinical skills. The benefits of being a simulation educator have been previously documented [17, 22].

The PGs expressed enthusiasm to become simulation educators. A previous study at MUST and other academic institutions in Uganda showed that PGs enjoy involvement in training undergraduate students [16]. This is similar to findings in another UK training region where core medical trainees expressed interest in teaching juniors [49] PGs also noted that they are suitable for the medical simulation facilitator role because of the already existing opportunity to teach undergraduate medical students. This is similar to findings from another previous study where the facilitator role was considered appropriate for PGs while the planner and resource developer roles were thought less suitable [20]. It is known that with formal training PGs are able to become competent medical simulation instructors [50]. The inclination of PGs to the educator role can be harnessed by building their capacity in simulation facilitation.

The heads of department and PGs were vaguely aware of the simulation educator role however, previous experience varied. The variation in experience among participants is likely due to difference in duration of exposure to SBL. MUST simulation center is one of the first in East Africa and already has alumni at postgraduate level who were exposed to SBL during their undergraduate program. Other academic institutions in the region are at different levels of implementing SBL with some having no simulation labs at all. Awareness of the role of a medical simulation facilitator is instrumental in establishing champions for the program [43].

### Recommendations

We recommend tailoring of PG simulation session to the different departmental schedules during future curriculum modification. Simulation educator training courses should be designed to fit conveniently in the departmental schedules. We recommend assessing the impact of PG simulation educators on undergraduate medical students’ performance to demonstrate productiveness of the program and increase its attractiveness. We recommend faculty engagement in mentoring of PG facilitated simulation sessions for sufficient role modeling and to encourage effort among the trained PG educators [36].

### Strengths and limitations

Our study has important strengths. First, we collected unique data from PGs medical trainees and this subgroup has important potential to contribute to SBL. We used different approaches to data collection including in-depth interviews, key informant interviews and focus group discussions. To reduce bias, the qualitative data collectors were not members of the simulation center and were not known to the participants. Another strength of the study is that we had an implementation framework to design and conduct the data collection.

Our study has some limitations. The study did not include undergraduate students to explore their perspectives on having PGs as their simulation educators. It would also be vital to explore the acceptability of the PG facilitators among undergraduate medical students. Another limitation is that this was a single site study and therefore, there may be limits to how the results from this study may be transferable to other settings. However, the results may have important lessons for institutions in similar settings such as MUST.

## Conclusion

In conclusion our study shows that involving PG as medical simulation educators is perceived to compliment bedside clinical teaching and to be beneficial to both undergraduate and postgraduate medical trainees. The PGs are enthusiastic to be trained as simulation educators, but desire SBL training within their curriculum. There is need to integrate simulation session by PGs into the timetables in the different departments to enable their participation without conflicting scheduling and competing time demands. Engaging the primary departments will promote ownership of the program.

## Data Availability

The transcripts generated and/or analyzed during this study are not publicly available to protect the participants’ confidentiality but are available from the corresponding author on reasonable request and with approval from the Research Ethics committee at Mbarara University of Science and Technology.

## Abbreviations

CFIR: Consolidated Framework for Implementation Research
FGD: Focused Group Discussion
HoD: Head of Department
IDI: In-depth Interview
KII: Key informant interview
MUREC: Mbarara University of Science and Technology Research Ethics Committee
MUST: Mbarara University of Science and Technology
PG: Postgraduate
PhD: Doctor of Philosophy
RADaR: Rigorous and Accelerated Data Reduction
RaT: Resident as Teacher
SBL: Simulation Based Learning
SIM for LIFE: Simulation for Life
UME: Undergraduate medical education
UNCST: Uganda National Council for Science and Technology
